# Integrating microalgae-based wastewater treatment, biostimulant production, and hydroponic cultivation: a sustainable approach to water management and crop production

**DOI:** 10.3389/fbioe.2024.1364490

**Published:** 2024-02-15

**Authors:** Ainoa Morillas-España, Raúl Pérez-Crespo, Silvia Villaró-Cos, Laura Rodríguez-Chikri, Tomas Lafarga

**Affiliations:** ^1^ Department of Chemical Engineering, University of Almeria, Almeria, Spain; ^2^ CIESOL Solar Energy Research Centre, Joint Centre University of Almeria-CIEMAT, Almeria, Spain

**Keywords:** waste management, raceway, photosynthesis, eutrophication, zucchini, agricultural products

## Abstract

A natural appearing microalgae-bacteria consortium was used to process urban wastewater. The process was done in an 80 m^2^ raceway reactor and the results were compared to an identical reactor operated using freshwater supplemented with commercial fertilisers. The biomass harvesting was done using commercial ultrafiltration membranes to reduce the volume of culture centrifuged. The membrane allowed achieving a biomass concentration of ∼9–10 g L^−1^. The process proposed avoids the use of centrifuges and the drying of the biomass, two of the most energy consuming steps of conventional processes. The specific growth rate in freshwater and the wastewater-based media was estimated as 0.30 ± 0.05 and 0.24 ± 0.02 days^−1^, respectively (*p* < 0.05). The maximum concentration reached at the end of the batch phase was 0.96 ± 0.03 and 0.83 ± 0.07 g L^−1^ when the biomass was produced using freshwater and wastewater, respectively (*p* < 0.05). The total nitrogen removal capacity of the system was on average 1.35 g m^−2^·day^−1^; nitrogen assimilation into biomass represented 60%–95% of this value. Furthermore, the P-PO_4_^3−^ removal capacity of the system varied from 0.15 to 0.68 g m^−2^·day^−1^. The outlet effluent of the reactor was used as a nutrient source in the hydroponic production of zucchini seedlings, leading to an increase in the root dry weight and the stem diameter compared to the water alone. The produced biomass showed potential for use as feedstock to produce plant biostimulants with positive effects on root development and chlorophyll retention.

## 1 Introduction

Wastewater generation is a critical issue that poses several challenges both for the environment and public health. Rapid growth of urban centres, industrialisation, and agricultural intensification has led to a significant increase in the volume of wastewater produced, which is expected to increase by 24% by 2030 and 51% by 2050 (Qadir et al., 2020). The incorrect management of wastewater contributes to pollution of our water bodies, deterioration of freshwater resources, and the spread of waterborne diseases (Abd-Elaty et al., 2022). Proper treatment and management of wastewater is not just an ecological concern, but also a complex logistic and infrastructure challenge. Wastewater treatment has played a key role in protecting public health and protecting the environment: It removes harmful pollutants, pathogens, and other contaminants, reducing the risk of waterborne disease and environmental degradation. However, conventional wastewater treatment processes can be energy- and resource-intensive and contribute to greenhouse gas emissions and resource consumption (Nguyen et al., 2019).

Several microalgae-based wastewater treatment plants have been constructed in the last decade, and many more are currently being built. By using microalgae, carbon dioxide is captured simultaneously with the nutrients from the wastewater and the biomass produced can be used as a source of bioenergy and other bioresources such as natural pigments (Maysarah Satya et al., 2023). Microalgae-bacteria consortia are efficient in recovering nutrients and reducing the chemical oxygen demand of wastewater. A symbiotic interaction occurs between microalgae and bacteria (Shiong Khoo et al., 2021). For example, algae supply oxygen for bacteria and bacteria supply carbon dioxide for microalgae. In addition, bacteria can excrete microalgae growth-promoting substances (Fallahi et al., 2021). One of the main bottlenecks of microalgae-based wastewater treatment is the need for large surface areas (Acién et al., 2016). The surface area required to process wastewater following a microalgae-based approach can be 30–50 times higher than that needed in conventional processes, where light availability is not a limiting factor (Acién et al., 2016). However, the many benefits of the process that include reduced greenhouse gas emissions, energy savings, decreased pollutant and pathogen levels, and the recovery of the nutrients in the form of microalgal biomass make the process more sustainable (Arashiro et al., 2018) and economically viable (Ansari et al., 2019). Due to the large surface area requirements, the implementation of microalgae-based wastewater treatment plants is limited to rural areas and small towns. One positive aspect is that the cost of conventional treatment plants in these locations is excessive, and they generally lack efficient water management systems (Acién et al., 2016). This strategy would also create more employment in rural areas and provide farmers with locally sourced agricultural inputs, as one of the main current applications of microalgae is the production of plant biostimulants and biopesticides (Morillas-España et al., 2022a).

The interest in plant biostimulants is growing and so is the popularity of algae-derived biostimulant extracts. Several research studies have demonstrated that algae have biostimulant effects that include improved germination, root development, fruit yields and fruit quality, amongst other benefits (Puglisi et al., 2020; Amaya-Santos et al., 2022; Gitau et al., 2022; Alling et al., 2023; Villaró et al., 2023). Most of these works were done at the laboratory scale or using specific microalgal strains. The goal of the present study was to process wastewater using a natural microalgae-bacteria consortium at a scale of 80 m^2^ and to evaluate the potential use of the produced biomass to obtain agricultural products with biostimulant effects. Typically, urban wastewater contains 20–35 mg L^−1^ of nitrogen (Li et al., 2017) and 6–12 mg L^−1^ of phosphorus (Di Capua et al., 2022). As the wastewater we used was collected from the University of Almeria (mainly blackwater), the total nitrogen and phosphorus content was relatively high, ∼55 and ∼30 mg L^−1^, respectively. For this reason, a secondary goal of this work was to evaluate the potential use of the treated water as a nutrient source for the hydroponic production of zucchini seedling. The process followed consisted of using ultrafiltration membranes to harvest and concentrate the biomass, avoiding the use of centrifuges that are energy consuming. The use of membranes in microalgae photobioreactors is gaining increased interest. One of their major benefits to the process is that they allow decoupling the hydraulic from the cellular retention time (Chaleshtori et al., 2022).

## 2 Materials and methods

### 2.1 Wastewater treatment

**FIGURE 1:**
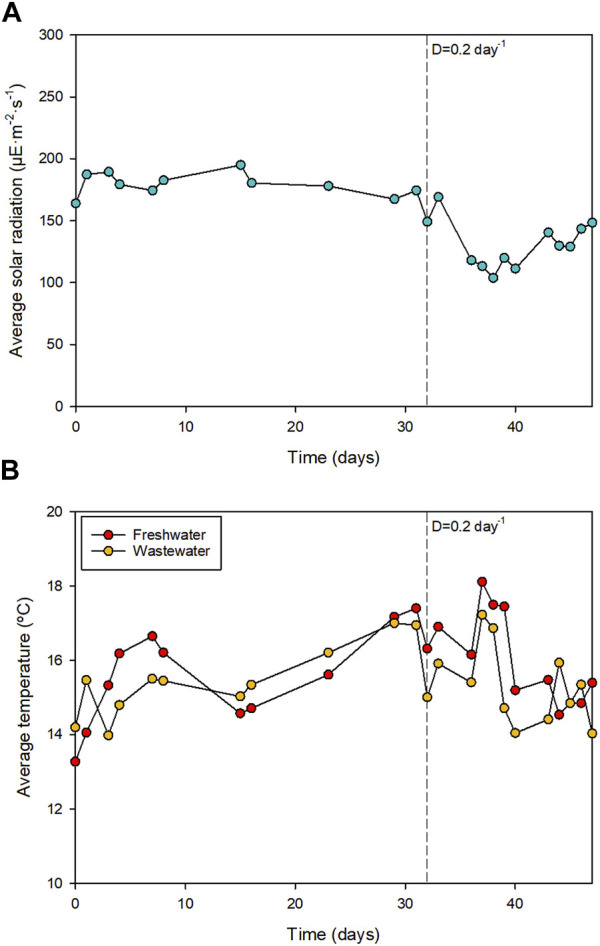
**(A)** Average solar radiation reaching the culture during the approximately 12 h of illuminated period and **(B)** Average temperature of the culture.

The wastewater treatment was done at the SABANA Demonstration Facility at IFAPA-University of Almeria (Spain). The average environmental conditions during the wastewater treatment are shown in Figure 1. The wastewater was processed using two identical 80 m^2^ raceway reactors located inside a greenhouse. These reactors are described in detail in a previous publication (Morillas-España et al., 2020). One of the reactors was operated using wastewater and the other was operated using freshwater supplemented with commercial fertilisers. The wastewater was collected directly from the University of Almeria and was not treated besides a filtration step where the solids were removed. The freshwater-based culture medium contained 0.90 g L^−1^ NaNO_3_, 0.18 g L^−1^ MgSO_4_, 0.14 g L^−1^ K_2_PO_4_, and 0.03 g L^−1^ of Karentol^®^ (Kenogard, Spain).

The reactors were operated at a culture depth of 0.15 m (12.8 m^3^). They were inoculated with the outlet effluent of a similar raceway reactor located outside the greenhouse, which had been operating in continuous mode for approximately 6 months and had an unknown microbial diversity (dominated by *Scenedesmus/Tetradesmus* strains based on microscope observations). This reactor was being operated using commercial fertilisers as the nutrient source and the concentration of the inoculum was 0.6 g L^−1^. The reactors were operated initially in batch mode until the biomass concentration was constant and then using a fixed dilution rate of 0.2 days^−1^ for 14 days. This mean that during these 14 days, 2.56 m^3^ of culture were harvested daily and replaced with fresh culture medium. The culture harvested was pre-concentrated using a 0.03 µm PULSION^®^ hollow fiber submerged membrane module (Kovalus Separation Solutions, MA, United States) and then centrifuged using an SSD 6-06-007 GEA separator (GEA Westfalia Separator Group, Germany).

### 2.2 Biomass processing

Part of the harvested biomass was frozen, freeze-dried, and stored at −20°C until further analysis. The remaining biomass was diluted until a concentration of 2 g L^−1^ prior to cell wall disruption using a UP400S ultrasonic processor (Hielscher Ultrasound Technology, Germany). The disruption efficiency was estimated by measuring the change in optical density during sonication as described elsewhere (Villaró et al., 2023) using a Genesys 10S UV–Vis spectrophotometer (Thermo Fisher Scientific, Spain).

### 2.3 Analytical determinations

The biomass concentration was calculated by filtering 50 mL of culture using pre-weighed and pre-dried 1 µm filters and drying in an oven at 70°C for 24 h. The biomass productivity was calculated as the product of the biomass concentration and the dilution rate, which was 0.2 day^−1^.

The macromolecular composition of the freeze-dried biomass (total proteins, total lipids, and ash content) was determined as described elsewhere (Ciardi et al., 2022). Briefly, the protein content was estimated based on the nitrogen content of the biomass determined using the Kjeldahl method. A nitrogen-to-protein conversion factor of 5.9 was used (Villaró et al., 2022). The lipid and ash contents were determined gravimetrically after extraction using chloroform:methanol (2:1, v/v) and after calcination at 600°C for 12 h, respectively. The concentration of carbohydrates was estimated by difference. The concentration of chlorophylls was estimated as described elsewhere (Ciardi et al., 2022) using a Genesys 10S UV–Vis spectrophotometer (Thermo Fisher Scientific, Spain). Furthermore, the total phenolic content of the biomass was estimated following the Folin Ciocalteu method as described elsewhere (Lafarga et al., 2018).

**FIGURE 2:**
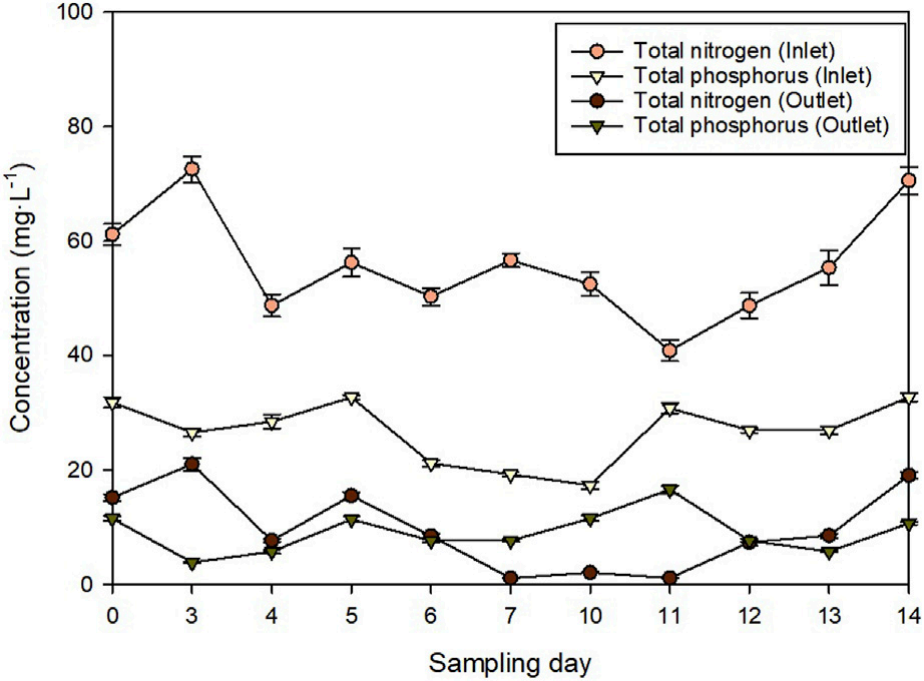
Daily nitrogen and phosphorus concentration of the wastewater before and after the microalgae-based treatment.

The concentration of N-NH_4_^+^, N-NO_2_^−^, N-NO_3_^−^, and P-PO_4_^3−^ was determined using standard methods described in previous works (Morillas-España et al., 2021c). Overall, the N-NO_2_^-^ and N-NO_3_^−^concentration was below 0.5 mg L^−1^. The N-NH_4_^+^ concentration ranged between 40 and 75 mg L^−1^ and the P-PO_4_^3-^ content was in the range 17–33 mg L^−1^. All the analytical determinations were done in triplicate and the results are shown as average value ±standard deviation. The daily inlet concentration of nitrogen and phosphorus is shown in Figure 2.

### 2.4 Assessment of biostimulant effects

The gibberellin-like effect of the biomass was evaluated by measuring the germination index of watercress seeds (*Lepidium sativum* L.). Briefly, 25 seeds were placed on Whatman No. 5 filter paper and put inside a sterile Petri dish. The Petri dishes were either treated with 2 mL of distilled water or microalgal extract in triplicate. The seeds were allowed to grow for 3 days in a controlled chamber at 24°C and the germination index was calculated as:

$$Germination\;index\;\left(\%\right)=\frac{N\cdot L}{N_{c}\cdot L_{C}}\cdot 100$$

where N is the number of germinated seeds, L is the average length of the germinated seeds and $N_{c}$ and $L_{C}$ are the number of germinated seeds and their length when only distilled water was added.

The root induction capacity was used to evaluate the auxin-like effect of the extracts. This was done using soybeans (*Glycine max* L.). The seeds were placed at a depth of 1 cm in moistened perlite and were left to grow during 7 days in a controlled chamber (27°C; 12/12 h light/dark cycle). Then, three seedlings were cut below the cotyledon, placed in vials containing 20 mL of the extract and incubated during 7 days in a controlled chamber (27°C; 12/12 h light/dark cycle). Three vials were prepared per treatment. The number of adventitious roots on each hypocotyl (larger than 1 mm) were counted and the results are expressed as the percentage of variation with respect to the samples treated with distilled water alone.

The cytokinin-like effect was determined using the excised cucumber (*Cucumis sativus* L.) expansion test. The seeds were placed on glass trays containing 0.7% agar-solidified Knop medium as described in a previous work (Morillas-España et al., 2022b). The trays containing the seeds were incubated for 5 days at 27°C in the dark. Then, 5 uniform cotyledons were weighed and transferred to Petri dishes containing a filter paper moistened using 3 mL of the test solutions. Four Petri dishes were prepared per treatment. The results are expressed in percentage of weight variation with respect to the samples treated with distilled water.

Finally, the chlorophyll retention assay was carried out using wheat (*Triticum aestivum* L.) seeds that were previously rinsed in tap water for 4 h. Then, the seeds were planted at a 1 cm depth in moistened perlite inside a controlled growth chamber (25°C, 65% relative humidity, 12/12 light/dark cycle). The leaves from the seedlings were cut in 10 mm segments; the first 3 cm from their apical tip were discarded. The fresh weight of ten segments was measured with an analytical balance and placed in 50 mL tubes containing 10 mL of either distilled water (the negative control) or the test sample. Three vials were used for each treatment. The vials were placed back inside the controlled chamber, where they remained for 4 days. Subsequently, the leaves were blot dried and put into graduated 15 mL tubes containing 8 mL of 80% ethanol in distilled water (v/v). The test tubes were transferred to a water bath at 80°C for 10 min and the solution was then chilled using an ice bath. The extracts were centrifuged, and the optical density determined at 645 nm using a spectrophotometer. The optical density was normalised to 100 mg fresh weight and the adjusted results were compared to the control, which was distilled water. Commercial hormones, namely, gibberellic acid (GA3), indol-3-butyric acid (IBA), and kinetin (KIN), were used as positive controls at a concentration of 1 mg·L^−1^. The microalgal extracts were assessed at concentrations of 2.0 g L^−1^, which was obtained directly after the cell wall disruption stage, and 0.5 g L^−1^, obtained by diluting the initial extract using distilled water.

The water effluents obtained from the biomass harvesting were used to produce zucchini (Curcubita pepo L.) seedlings using a Duronic GHS37 system (Duronic, United Kingdom) located in a room with the temperature controlled at 22°C ± 2°C and a 12:12 h photoperiod. The seedlings were initially sowed on turf at a controlled chamber (24°C ± 1°C, 73% ± 5% RH) for 7 days and then placed in the hydroponic system. The results of the process waters were compared to those obtained using tap water alone and irrigation water containing 0.90 g L^−1^ NaNO_3_, 0.18 g L^−1^ MgSO_4_, 0.14 g L^−1^ K_2_PO_4_, and 0.03 g L^−1^ of Karentol^®^ (Kenogard, Spain). After 25 days, different parameters including the root’s dry weight, the roots length, the diameter of the stem, the foliar area of the first and second leaves, and their colour were determined. Eight plants were used per treatment.

### 2.5 Statistical analysis

Data were analysed using either a *t*-test, to assess differences between means of two variables, or an ANOVA, to assess differences between the means of three or more. Subsequently, a Tukey HSD test was carried out to find where the sample differences occurred and the criterion for statistical significance was *p* < 0.05. These statistical procedures were done using Statgraphics 18 (Statgraphics Technologies Inc., United States).

## 3 Results and discussion

### 3.1 Wastewater treatment and biomass production

**FIGURE 3:**
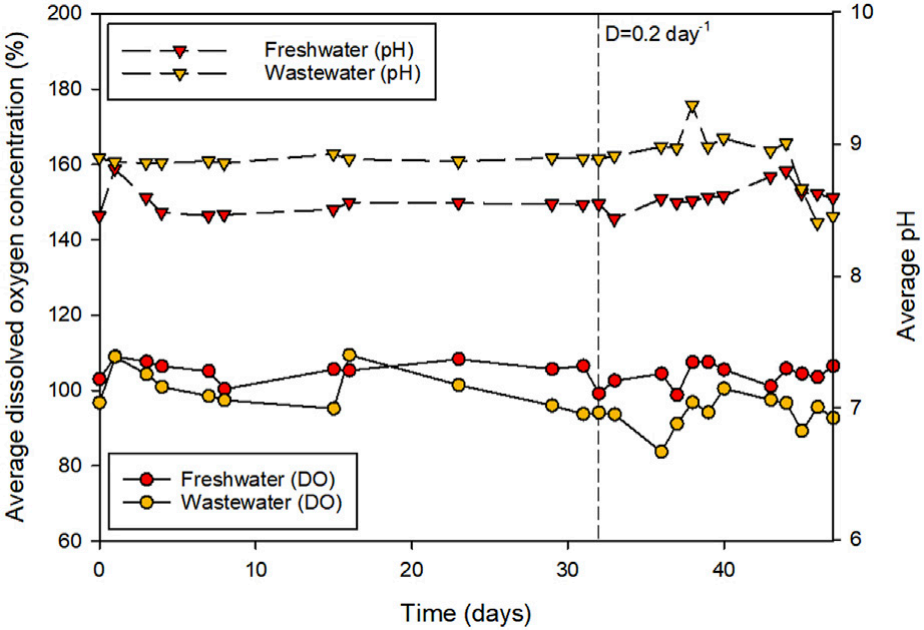
Dissolved oxygen concentration and pH of the cultures during the biomass production.

The environmental conditions during wastewater treatment are shown in Figure 1. In general, the average irradiance that reached the surface of the water was 147.7 ± 32.7 μE m^-2^·s^−1^, with maximum daily values of 1420.3 ± 129.4 μE m^−2^·s^−1^. The daily maximum, minimum, and average temperatures inside the greenhouse were 19.6 ± 1.9, 11.5 ± 2.7, and 15.8°C ± 1.9°C, respectively. Figure 3 shows the concentration of dissolved oxygen (DO) and the pH during the batch and the semi-continuous phases of production. Previous works demonstrated that the high growth rate of microalgae can lead to the accumulation of DO at concentrations that inhibit their growth (Morillas-España et al., 2020). In this work, DO concentrations were adequately controlled with average values of around 100%. This was achieved by continuous injection of air at a flow rate of 200 L min^-1^ in the reactor. The presence of aerobic bacteria in the wastewater could also have contributed to the controlled oxygen concentration in the medium. The pH was also adequately controlled with average values ranging from 8.5 to 9.0.

**FIGURE 4:**
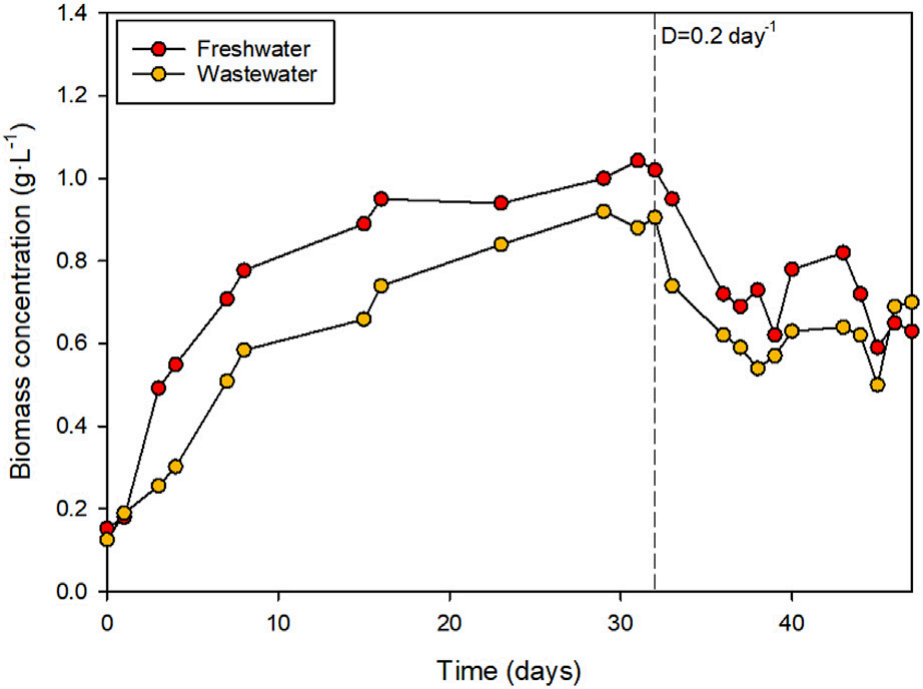
Biomass production using freshwater supplemented with synthetic nutrients or wastewater.

The type of water used affected the growth of the microalgae. The results, shown in Figure 4, demonstrated a higher growth rate in the freshwater-based medium than in the wastewater (*p* < 0.05). The specific growth rate in freshwater and the wastewater-based media was estimated as 0.30 ± 0.05 and 0.24 ± 0.02 day^−1^, respectively (*p* < 0.05). The higher growth rate can be partially attributed to the adaptation to the new culture medium, as the inocula were obtained from a photobioreactor operated using freshwater supplemented with fertilisers. The maximum concentration reached at the end of the batch phase was 0.96 ± 0.03 and 0.83 ± 0.07 g L^−1^ when the biomass was produced using freshwater and wastewater, respectively (*p* < 0.05). The biomass productivity during the semi-continuous production stage was 21.0 ± 1.8 and 18.8 ± 2.4 g m^−2^·day^−1^ when the biomass was produced using freshwater and wastewater, respectively. No statistical differences were observed between these values (average 19.8 g m^−2^·day^−1^). The biomass production capacity of the system was in line with that reported in previous work where microalgal biomass was produced using wastewater. For example, biomass productivity values of 17.0 g m^−2^·day^−1^ (Posadas et al., 2015) and 20–30 g m^−2^·day^−1^ (Sánchez-Zurano et al., 2021) have been reported when producing biomass using wastewater in raceway reactors. The results are difficult to compare, as the productivity of photobioreactors depends on their design, location (environmental conditions), microbial diversity, wastewater composition, and operational conditions. Furthermore, no differences in the Fv/Fm value of the cultures were observed, which is a measure of the efficiency of photosynthetic electron transport. Differences in this value may indicate stress or damage to the photosynthetic apparatus caused by nutrient deficiencies, photoinhibition, high temperature, or the presence of toxic compounds The data suggested that the cultures were not stressed with an average value of 0.62 ± 0.09 during the semi-continuous production phase.

**FIGURE 5:**
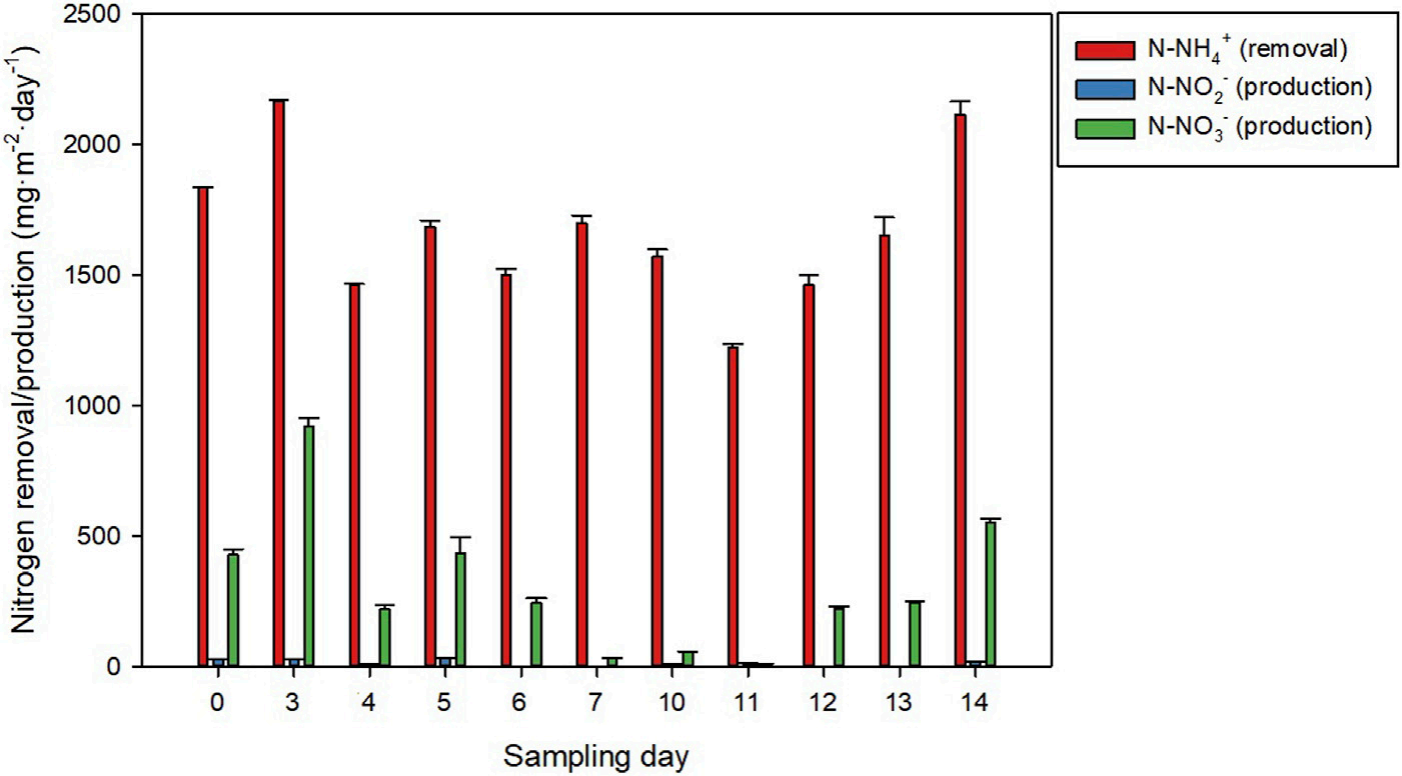
Nitrogen recovery capacity of the system.

One of the challenges of treating wastewater using microalgae is that its composition is highly variable between seasons and even within weeks or days. For this reason, robust strains that are capable to cope with different nutrient concentrations should be favored. In this work, for example, the main nitrogen source in the wastewater was N-NH_4_^+^, with inlet concentrations ranging from 40 to 75 mg L^−1^. Figure 5 shows the daily total nitrogen recovery capacity of the system during the semi-continuous production stage. The N-NH_4_^+^ removal capacity of the process was 100%, with areal removal rates ranging from 1.4 to 2.2 g m^−2^·day^−1^. The concentration of N-NO_2_^−^ and N-NO_3_^−^ in the inlet effluents was almost negligible, with maximum concentrations of 0.3 mg L^−1^ and most of the days being below 0.05 mg L^−1^. The opposite was observed in the outlet effluents, where the maximum N-NH_4_^+^ concentration was 0.3 mg L^−1^ and both N-NO_2_^−^ and especially N-NO_3_^−^ were the predominant nitrogen sources. The results revealed that the N-NH_4_^+^ removed from the wastewater during the process was not just assimilated by microalgae into biomass but also transformed into N-NO_2_- and N-NO^3−^ by nitrifying bacteria. This phenomenon is common and has been reported in previous work (Morillas-España et al., 2021b). While microalgae use N-NH_4_^+^ to produce more biomass, nitrifying bacteria use it as a source of electrons and oxidise it to NO_2_^−^ and NO_3_^−^. A recent publication reviewed this phenomenon and reported that nitrification can represent 15%–70% of the total N-NH_4_^+^ removed in microalgal cultures (González-Camejo et al., 2022). In this work, on average, the amount of N-NH_4_^+^ that was transformed into N-NO_2_^−^ and N-NO_3_^−^ was 17.6% although a high variability was observed between the different sampling days. The reason for this is that the factors affecting the competition for N-NH_4_^+^ include temperature and light and these are highly variable (Vergara et al., 2016). Overall, a mass balance of the system revealed that the total nitrogen removal capacity of the system was on average 1.35 g m^−2^·day^−1^. Nitrogen assimilation into biomass represented 60%–95% of the total nitrogen that entered the reactor. The outlet effluent contained 1%–40% of the total nitrogen that entered the system, being the average 16.8%. In addition, no loss of nitrogen via volatilization was observed, except for 2 days when 5%–10% of the total nitrogen that entered the reactor was lost into the atmosphere. This demonstrates the good pH control of the process as technically, ammonia stripping requires pH values higher than 9.25 (Wu and Vaneeckhaute, 2022).

**FIGURE 6:**
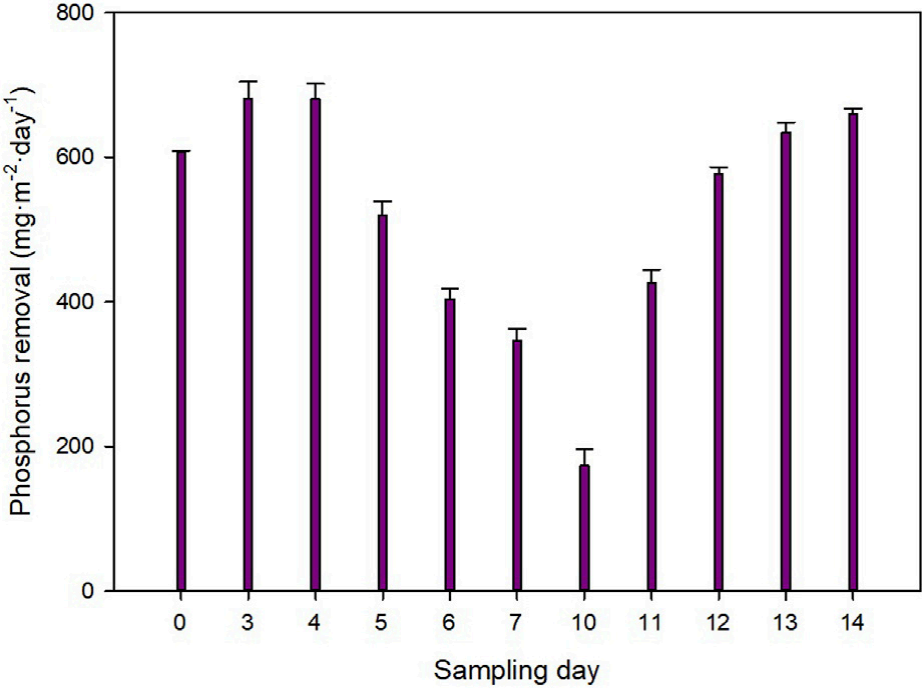
Phosphorus recovery capacity of the system.

Not just nitrogen but also phosphorus plays a key role in eutrophication. Figure 6 shows the total phosphorus recovery capacity of the system. Overall, the P-PO_4_^3−^ removal capacity of the system varied from 0.15 to 0.68 g m^−2^·day^−1^. The P-PO_4_^3−^ removal capacity of the system was higher than that reported for other microalgae such as *Anabaena* sp. and *Dolichospermum* sp. (Morillas-España et al., 2021a). Assuming a biomass phosphorus content of 2%, 30%–65% of the total phosphorus that entered the system was assimilated into biomass, while 20%–70% was still present in the outlet effluent. This is just an estimation, as it is known that microalgae can uptake more phosphorus than they need for survival when they are exposed to high phosphorus concentrations, as it happens in this work (Su, 2021). This phenomenon is known as “luxury uptake.” The main reason for the high phosphorus concentration in the outlet effluent is the high concentration in the inlet (Figure 2). Although the normal concentration in urban wastewater is 6–12 mg L^−1^ of phosphorus (Di Capua et al., 2022), in this study, the concentration ranged from 17 to 33 mg L^−1^. These high concentrations of phosphorus, together with a high variability of the composition of the wastewater, end up with an imbalance between the nitrogen and phosphorus ratios required for optimal growth and recovery of nutrients. Although the Redfield ratio (16:1, N:P) is often considered a fundamental principle in ecology, research has shown that this value can vary depending on the microalgal strain (Townsend et al., 2008). Despite the precision or not of this ratio, what all the optimal N:P molar ratios reported to date have in common is that the concentration of nitrogen is much higher than that of phosphorus. Because of the high phosphorus content of the wastewater, the N:P molar ratio in this study was on average 4.7 ± 1.2. This is the main reason why the outlet effluent contained such a high concentration of P-PO_4_^3−^ (Figure 2). To avoid this problem, the wastewater could be supplemented with nitrogen and/or the dilution rate lowered resulting in a higher hydraulic retention time. Nitrogen supply was effectively used to improve phosphorus removal in a previous publication (Beuckels et al., 2015).

### 3.2 Biomass processing

The cell wall of microalgae is diverse; while some species lack a rigid cell wall, others have resistant cell walls containing silica frustules (e.g., diatoms), cellulosic compounds (e.g., *Chlorella*), complex polysaccharides (e.g., *Tetraselmis*), or complex proteinaceous coverings (e.g., *Euglena*) (Alhattab et al., 2019). To recover the bioactive or functional compounds produced by microalgae or to maximise their availability by plants or animals, the disruption of the cell wall is not optional (Teuling et al., 2019; Amaya-Santos et al., 2022). In this work, the cell wall disruption was done by sonication at a concentration of 2 g L^−1^. The disruption was estimated as the optimal when the absorbance (680 nm) of the culture medium stopped increasing. This was achieved after 6 min of sonication in both cases. The disrupted biomass was evaluated as a plant biostimulant. The results are shown in the following section. In this study, the biomass was evaluated as a source of plant biostimulants.

The use of biomass following a biorefinery approach can also be studied. For example, *Arthrospira platensis* was used as a protein source of proteins and the leftovers from protein extraction were studied as plant biostimulants, obtaining better results when compared to the whole biomass (Villaró et al., 2023). Future work will evaluate the potential extraction of a valuable product (e.g., proteins, pigments, or lipids) before the formulation of the biostimulant extract. The macromolecular composition of the biomass is listed in Table 1. In general, no differences were found between the protein and ash content of the biomass produced using freshwater or wastewater. However, biomass production using wastewater led to higher concentration of total phenolics and lipids and lower chlorophyll and carbohydrate contents (*p* < 0.05). The protein content of the biomass was especially high, representing almost 50% of the total dry weight. Protein hydrolysates are known biostimulant extracts that can increase agricultural yields and fruit quality by enhancing nutrient uptake and abiotic stress tolerance (Colla et al., 2015). The high protein content of the biomass also suggests its potential use in the production of animal feeds, for example, aquafeeds (Bongiorno et al., 2020). The culture medium also affected the carotenoid concentration of the biomass. In particular, the concentration of fucoxanthin, neoxanthin, and α-carotene was higher (*p* < 0.05). These results also suggest the potential utilisation of the biomass as a natural feed additive, as the supplementation of aquafeeds with antioxidants is becoming essential not just to minimise oxidative stress but also to increase the stability and storage time of the product (Aklakur, 2018).

**TABLE 1:** Composition of the produced biomass. Values represent the mean of three independent determination ±standard deviation.

Compound	Freshwater	Wastewater
Protein (g·100 g^−1^)	47.1 ± 2.9^A^	45.9 ± 3.1^A^
Lipid (g·100 g^−1^)	3.9 ± 0.2^B^	9.1 ± 0.4^A^
Carbohydrate (g·100 g^−1^)	40.3 ± 0.7^A^	36.3 ± 0.9^B^
Ash (g·100 g^−1^)	8.9 ± 0.3^A^	8.7 ± 0.3^A^
Chlorophylls (mg·100 g^−1^)	51.2 ± 6.2^A^	40.6 ± 3.3^B^
Total phenolic content (mg·100 g^−1^)	0.21 ± 0.09^B^	0.36 ± 0.02^A^
Fucoxanthin (mg·kg^−1^)	0.05 ± 0.00^B^	0.37 ± 0.04^A^
Neoxanthin (mg·kg^−1^)	0.09 ± 0.01^B^	0.42 ± 0.01^A^
Violaxanthin (mg·kg^−1^)	0.05 ± 0.00^A^	0.05 ± 0.01^A^
Lutein (mg·kg^−1^)	1.49 ± 0.03^A^	1.18 ± 0.07^B^
α-Carotene (mg·kg^−1^)	0.13 ± 0.00^B^	0.29 ± 0.05^A^
β-Carotene (mg·kg^−1^)	15.21 ± 0.92^A^	14.92 ± 0.53^A^

Different letters in the same line indicate statistical significant differences (*p*< 0.05).

### 3.3 Agricultural applications

**FIGURE 7:**
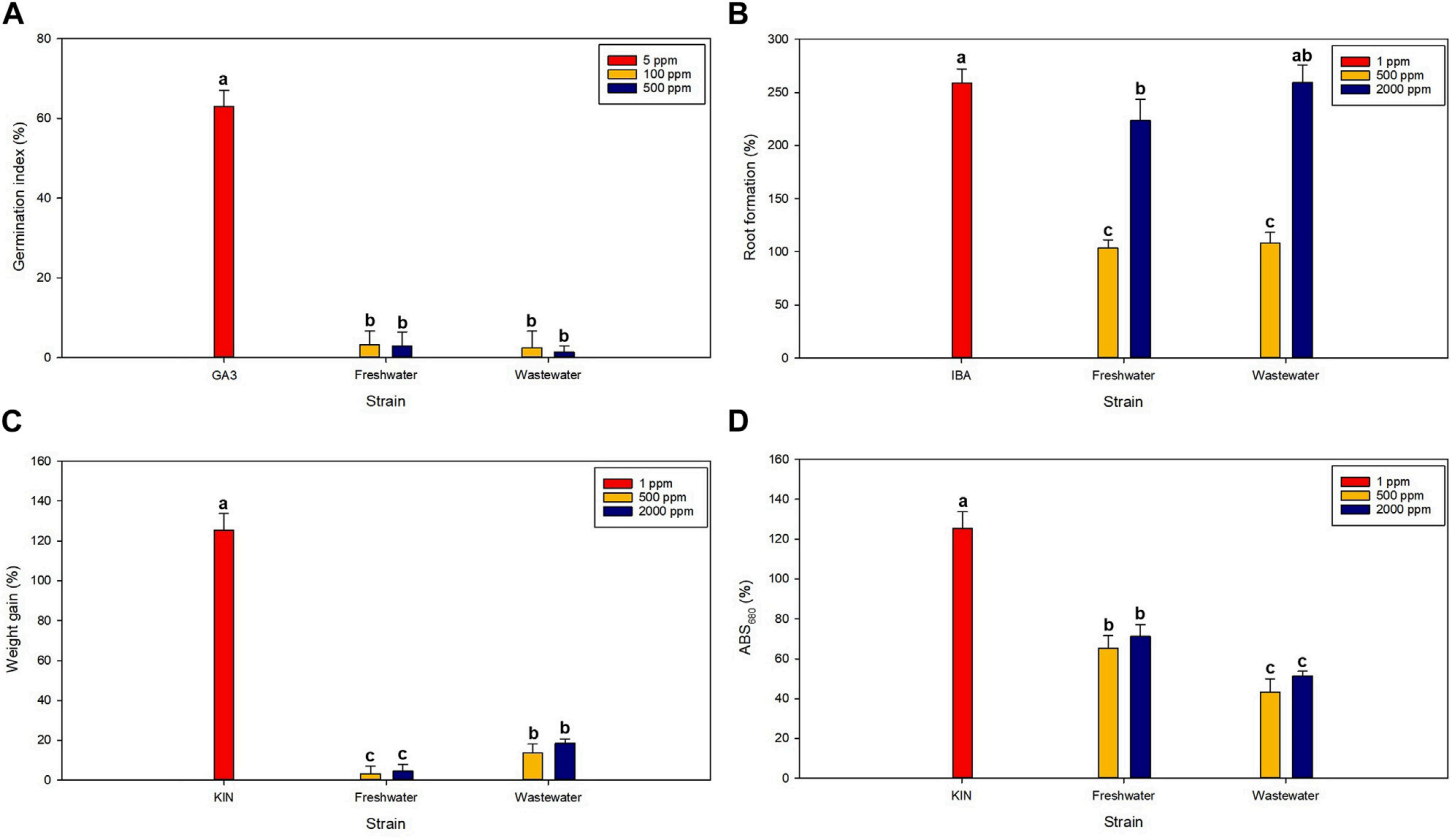
Biostimulant effects: Effect of microalgal biomass on the **(A)** germination index, **(B)** root formation, **(C)** weight gain, and **(D)** chlorophyll retention. The values represent the percentage of variation with respect to distilled water. Different letters indicate statistical differences (*p* < 0.05).

The biostimulant properties of the biomass produced were evaluated following *in vitro* bioassays and the results are shown in Figure 7. The first trial assessed the effect of the extracts on the germination index. The positive control used was gibberellic acid (GA3), which plays key roles in many essential plant development processes including germination (Ma et al., 2018). Gibberellins act through the activation of embryo growth, mobilization of reserves, and weakening of the endosperm layer (Ma et al., 2018). In this work, the germination index of the seeds was not improved after the application of none of the biomasses. The results are expressed as a percentage of increase with respect to distilled water alone, which means that the biomass did not affect the germination index. This contrasts with previous reports where the seed germination index was either negatively (Morillas-España et al., 2022b) or positively (Garcia-Gonzalez and Sommerfeld, 2016) affected by microalgal biomass. A recent work demonstrated that the degree of hydrolysis of the cell wall influenced the capacity of microalgae-derived compounds to promote germination (Navarro-López et al., 2020) and other works revealed that the effect depends on strain (Morillas-España et al., 2022b). While gibberellins are the main hormones related with initiation seed germination, auxins are the ones related with root development. Auxins constitute a group of low-molecular weight molecules having growth-inducing effects; they are naturally present in plants and are involved in several biological processes. Some of the most common auxins are inoleacetic acid (IAA) and indole-3-butyric acid (IBA) being the latter more effective in promoting root formation because of its higher stability (Gomes and Scortecci, 2021). The auxin-like activity, the capacity of the extracts to promote root development, was also evaluated. The results, shown in Figure 7, revealed that the biomass promoted root formation with approximate improvements of 100% and 240% when applied at a concentration of 0.5 and 2.0 g L^−1^, respectively. At the highest concentration studied, the microalgal biomass produced using wastewater showed a higher biostimulant effect (*p* < 0.05). The results were comparable to those obtained for *Chlorella vulgaris* produced using freshwater and wastewater (Amaya-Santos et al., 2022). Roots not only provide the anchor required to keep plants in place, but also act as the lifeline of plants taking up air, water, and nutrients. The use of plant biostimulants based on microalgae can improve root development and this in turn can boost nutrient uptake, growth, and adaptability to stress conditions. For example, microalgae-based extracts demonstrated to promote the adaptability of plants to saline conditions (Mutale-joan et al., 2021) and drought stress (Kusvuran, 2021) in previous years. The capacity of the extracts to improve weight gain and chlorophyll retention were also studied. Cytokinins are a group of hormones that are derived from adenine and participate in the regulation of growth, plant physiological activities, and in the plant’s response to abiotic stresses. Kinetin (KIN) was the first cytokinin discovered and plays roles in cell division, photosynthesis, nutrient metabolism, and maintenance of meristem function (Li et al., 2021). In this study, no major positive effects on the weight gain were observed, besides a 15%–20% increase when applying the biomass produces using wastewater at a concentration of 2 g L^−1^ (*p* < 0.05). The weight gain was higher when applying the biomass produced using wastewater. This can be attributed to the effect that the different compounds present in the wastewater have on the biomass composition; for example, a recent work revealed that wastewater can trigger the production of essential amino acids and carotenoids (Villaró-Cos et al., 2024). In addition, the chlorophyll retention test revealed that both extracts permitted a higher chlorophyll content when compared to distilled water (*p* < 0.05). The effect was higher in the biomass produced using freshwater (*p* < 0.05). Similar results were obtained when applying different microalgae, for example, the use of *C. vulgaris* increased the chlorophyll and carotenoid content of the edible part of lettuce seedlings (Puglisi et al., 2022). Previous work also reported an increased growth and chlorophyll content in plants treated with microalgae attributed to an improved osmotic adjustment (Mutale-joan et al., 2021) and to the protection of PSII (Gitau et al., 2022).

**FIGURE 8:**
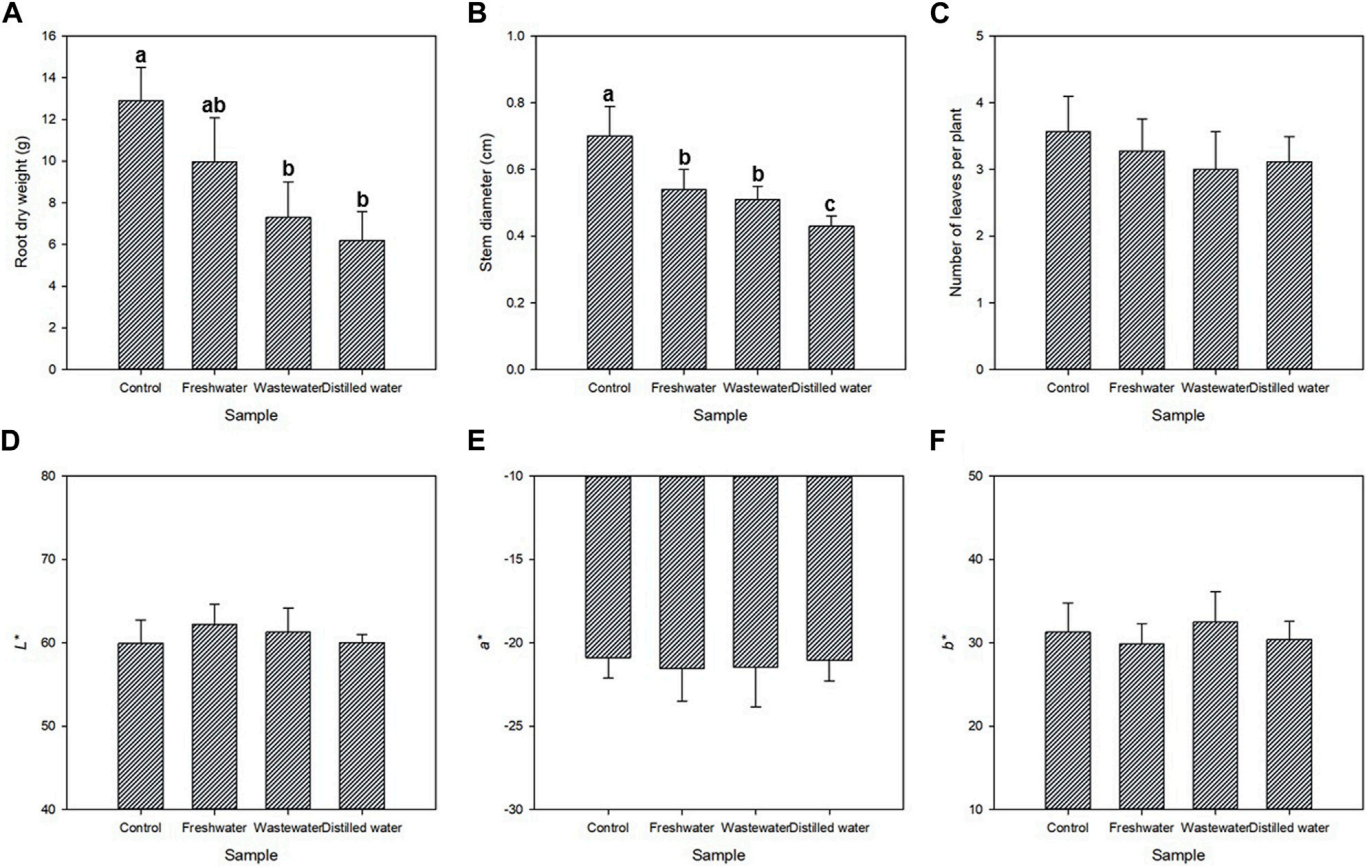
Water reuse: Effect of the process water on the **(A)** Root dry weight, **(B)** Stem diameter, **(C)** Number of leaves and the **(D)** L*, **(E)** a*, and **(F)** b* values of the zucchini seedlings. Different letters indicate statistical differences (*p* < 0.05).

One last objective of this study was to evaluate the potential use of the processed wastewater as a nutrient source in hydroponic cultivation of zucchini seedlings. The nitrogen and phosphorus content of the outlet effluents of the reactors is shown in Figure 6. Both were compared to a medium containing a standard recipe and distilled water. Overall, the seeds were able to germinate and grow in both the treated wastewater and the freshwater effluent where the microalgae were produced (Figure 8). In both cases, the root dry weight and the stem diameter was lower than in the standard medium, but higher than when using distilled water alone (*p* < 0.05). It is likely that supplementation of nutrients might be required in future work. However, the results suggest the potential use of these effluents in agriculture. The results presented herein are a preliminary approach that needs to be further optimized and validated. Moreover, no differences were observed in the colour of the seedlings nor in the number of leaves per plant compared to the standard recipe. The use of the outlet effluents of wastewater treatment photobioreactors in agriculture are a promising strategy to further recover the nutrients that were not consumed by microalgae and to reduce the freshwater requirements of agriculture.

## 4 Conclusion

A microalgae-bacteria consortium was used to recover nutrients from wastewater. The results presented here demonstrate that natural microalgal blooms can be used to recover nutrients from wastewater as the consortium was efficient in recovering nitrogen and phosphorus. However, due to the high nutrient load in the wastewater, the processed water still contained relatively high contents of nitrogen, mainly N-NO_3_^−^, and phosphorus. The treated wastewater showed potential for use as a nutrient source for the hydroponic production of zucchini seedlings. In addition, the biomass produced showed potential biostimulant effects. The process used consisted of concentrating the culture using ultrafiltration membranes and disrupting the cells using ultrasounds. No drying or centrifuging steps were required, rendering the energetic requirements low. Future work will evaluate different disruption strategies (e.g., high pressure homogenisation) and fractionate the biomass to identify which fraction and which compounds were the responsible for the observed biostimulant effects. The use of microalgae-bacteria consortia that grow naturally is the most realistic option for processing wastewater in open reactors and more work should be done to assess their potential applications.

## Data Availability

The raw data supporting the conclusion of this article will be made available by the authors, without undue reservation.
